# Supplementary material for: Comparative Analysis of the Antioxidant, Antidiabetic, Antibacterial, Cytoprotective Potential and Metabolite Profile of Two Endophytic Penicillium spp
Source: Antioxidants (Basel). 2023 Jan 22;12(2):248. doi: 10.3390/antiox12020248 (PMC9951918; doi:10.3390/antiox12020248)
Supplement: Supplementary file 1 [file antioxidants-12-00248-s001.zip › antioxidants-2143177-supplementary.pdf]

Comparative Analysis of the Antioxidant, Antidiabetic, Antibacterial, Cytoprotective Potential and Metabolite Profile of Two Endophytic *Penicillium* spp.

Kumar Vishven Naveen, Kandasamy Saravanakumar, Anbazhagan Sathiyaseelan, and Myeong-Hyeon Wang*

Department of Bio-Health Convergence, Kangwon National University, Chuncheon, 24341, Republic of Korea.

* Corresponding author.

Myeong-Hyeon Wang

Email: mhwang@kangwon.ac.kr

Materials and methods

Assessment of total phenolics and flavonoid content

The total phenolic content (TPC) in EAE-PL and EAE-PR was ascertained by the Folin-Ciocalteu's method. Briefly, 100 µL of test samples (EAE-PL and EAE-PR) in known concentration (1 mg/mL) was added with the 200 µL of Folin-Ciocalteu's phenol reagent, subsequently added with 500 µL of Na_2CO_3, and mixed well by gentle shaking. The reaction mix was incubated for 50 min at 25 ˚C and then read by a UV-vis spectrophotometer at 760 nm wavelength. The absorbance is recorded, the TPC is determined, and expressed as mg of GAE/g DW (gallic acid equivalent per gram of dry weight).

The total flavonoid content (TFC) in EAE-PL and EAE-PR was ascertained by the aluminum chloride method. In brief, 100 µL of test samples (EAE-PL and EAE-PR) in known concentration (1 mg/mL) was diluted with 250 µL of distilled water and added with 150 µL of EtOH (95%). Furthermore, added with 25 µL of $AlCl_3$ (10%) and 25 µL of CH_3COOK (1M) and then mixed well by gentle shaking. The reaction mix was incubated for 30 min at 25 ˚C and then read by a UV-vis spectrophotometer at 415 nm wavelength. The absorbance is recorded, the TFC is determined, and expressed as mg of QE/g DW (quercetin equivalent per gram of dry weight).

Alpha-amylase and alpha-glucosidase inhibition assay

In α-amylase inhibition assay, the 50 µL of EAE-PL and EAE-PR in serially diluted concentration (7.8-1000 µg/mL) was separately added to the 50 µL of α-amylase (2 Unit) and further mixed with 100 µL of phosphate buffer (1 mM, 6.9 pH). Acarbose was used as experimental positive control. The reaction mix was incubated for 15 min at 37 ± 1 °C, added with 50 µL of starch solution (0.5%), and further incubated for 15 min at 37 ± 1 °C. Following incubation, 500 µL of DNS reagent (0.1 M) was added to the reaction mix and incubated in a water bath for 5 min at 95 °C. Then, the reaction mix was diluted 5 folds and read spectrophotometrically at 540 nm of UV absorbance, and α-amylase inhibition (%) was calculated. The experimental groups without samples were taken as control.

In α-glucosidase inhibition assay, the 50 µL of EAE-PL and EAE-PR in serially diluted concentration (7.8-1000 µg/mL) was separately added to the 25 µL of α-glucosidase (1 Unit) and

further mixed with 100 µL of phosphate buffer (1 mM, 6.9 pH). Acarbose was used as experimental positive control. The reaction mix was incubated for 20 min at 37 ± 1 °C, added with 50 µL of PNPG solution (5 mM), and further incubated for 20 min in dark at 37 ± 1 °C. Following incubation, the reaction was stopped by adding 100 µL of Na_2CO_3 (0.1 M), and reaction mix was read spectrophotometrically at 405 nm of UV absorbance. PBS (6.9 pH) was used as experimental control to calculate the % of α-glucosidase inhibition.

$ABTS^+$ and DPPH radicals scavenging assay

In $ABTS^+$ radical scavenging assay, the $ABTS^+$ stock solution was prepared by mixing the ABTS (7 mM) and potassium persulphate (2.45 mM) in a 1:0.5 ratio and incubating overnight at room temperature in dark. Then, $ABTS^+$ radicals (working solution) were prepared by diluting the stock solution with EtOH (50%) and the absorbance was adjusted to 0.7 ± 0.02 at 734 nm. For $ABTS^+$ radical scavenging experiment, 100 µL of serially diluted concentration (7.8-1000 µg/mL) of EAE-PL and EAE-PR was separately added with the $ABTS^+$ working solution (100 µL). Meanwhile, serially varied concentration (7.8-1000 µg/mL) of standard AA and $ABTS^+$ mixed with methanol (without samples) were taken and experimental positive and negative control, respectively. The reaction mix was incubated for 10 min in the dark at room temperature. Finally, the absorbance of the reaction mix was recorded at 734 nm and, the % of $ABTS^+$ radical scavenging activity was calculated.

In DPPH radical scavenging assay, The DPPH (0.1 mM) was diluted in methanol (100 mL) to prepare the DPPH working solution. Then, 100 µL of serially diluted concentration (7.8-1000 µg/mL) of EAE-PL and EAE-PR was separately added to the 100 µL of DPPH solution. Meanwhile, the AA (experimental standard) was taken in the same concentrations to compare the results. The reaction mix was incubated for 15 min in the dark at room temperature. The DPPH added with methanol (without samples) was considered as experimental control. Finally, the absorbance of reaction mix was read at 517 nm spectrophotometrically (UV-vis spectrophotometer) and DPPH free radical scavenging percentage (%) was computed.

Results

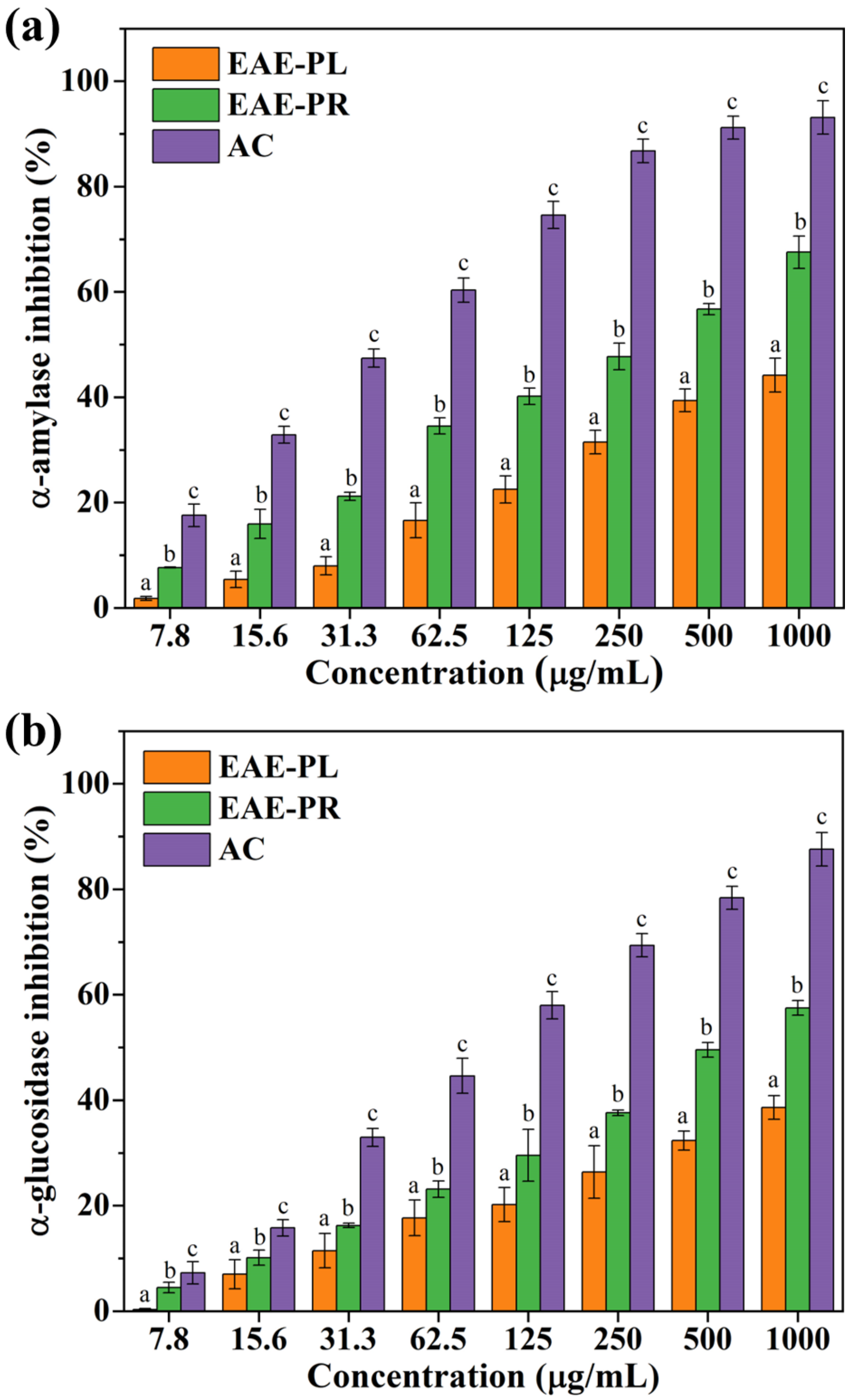
(a)
EAE-PL
EAE-PR
AC
α-amylase inhibition (%)
Concentration (μg/mL)
0
20
40
60
80
100
7.8
15.6
31.3
62.5
125
250
500
1000
(b)
EAE-PL
EAE-PR
AC
α-glucosidase inhibition (%)
Concentration (μg/mL)
0
20
40
60
80
100
7.8
15.6
31.3
62.5
125
250
500
1000

Figure S1. Analysis of α-amylase inhibition (a) and α-glucosidase inhibitory effect (b) of EAE-PL and EAE-PR compared to standard acarbose (AC). Error bars represent the SD of 3 independent experiments and alphabets at error bars represent significance ($p<0.05$) among the groups.

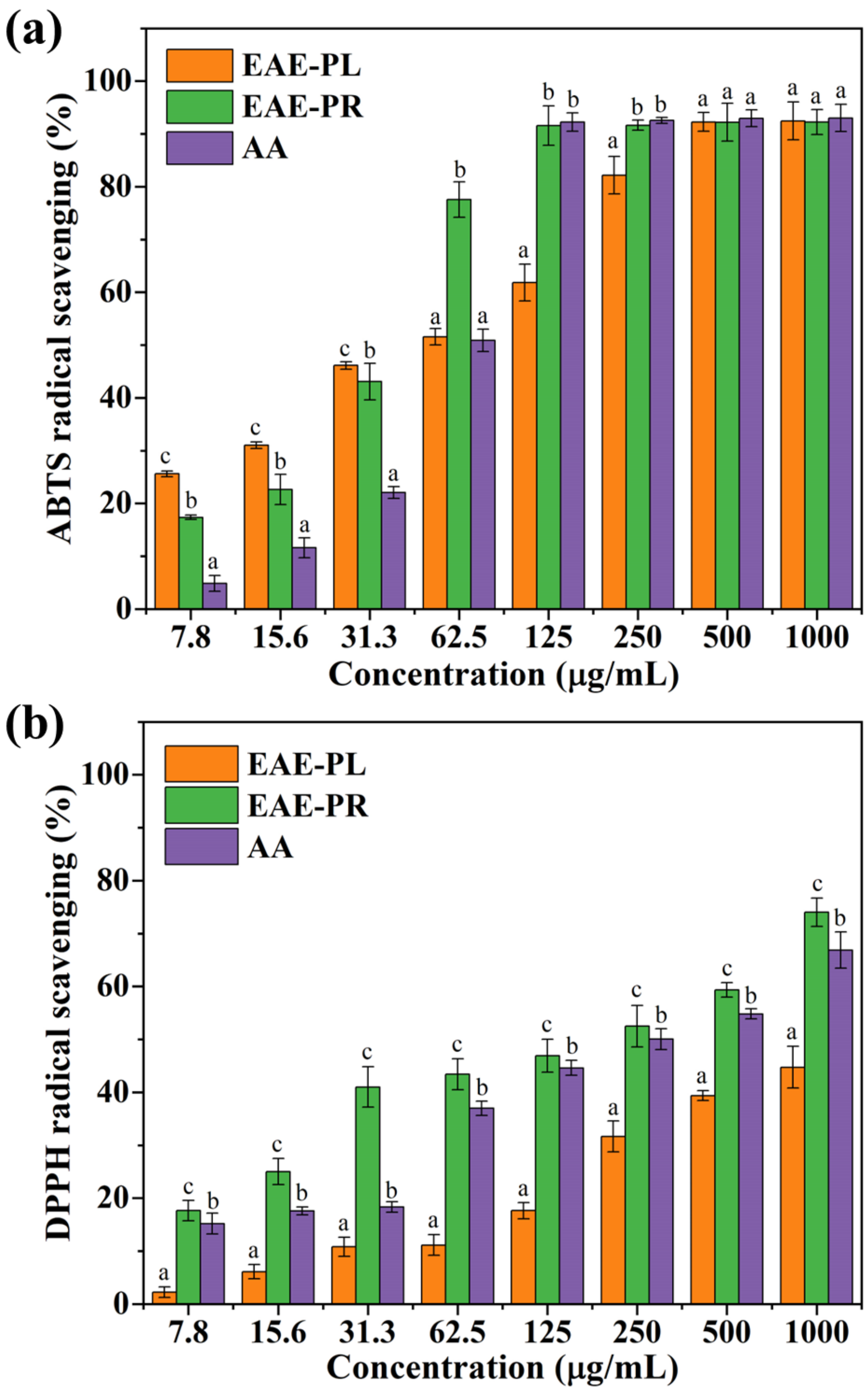

Figure S2. ABTS radical scavenging activity (a) and DPPH radical scavenging activity (b) of EAE-PL and EAE-PR compared to standard ascorbic acid (AA) during *in-vitro* free radical scavenging assays. Error bars represent the SD of 3 independent experiments and alphabets at error bars represent significance ($p<0.05$) among the groups.

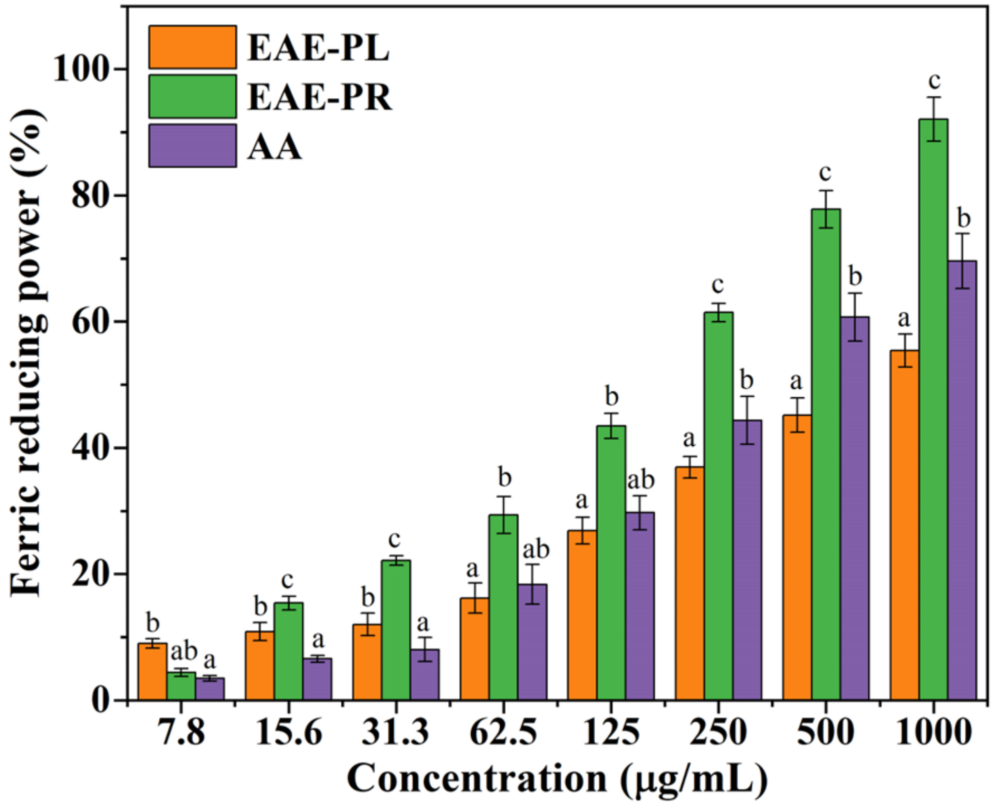

Figure S3. Ferric reducing antioxidant power of EAE-PL and EAE-PR compared to standard ascorbic acid (AA). Error bars represent the SD of 3 independent experiments and alphabets at error bars represent significance ($p<0.05$) among the groups.

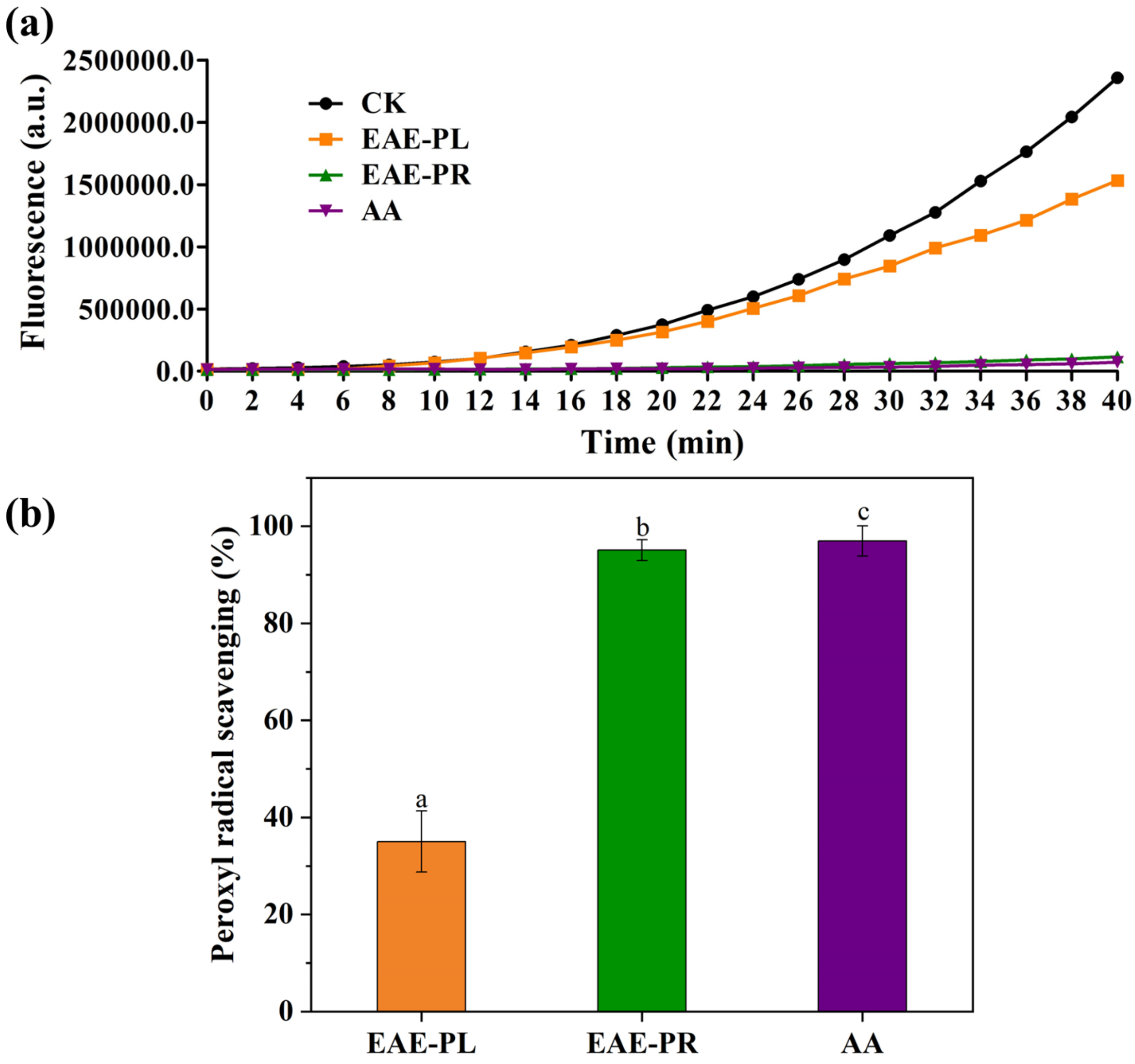

Figure S4. The peroxyl radical scavenging activity (PSC) of EAE-PL and EAE-PR compared to standard ascorbic acid (AA) analyzed at every second minute up to forty minutes (a) and analysis total PSC (%) at 40th minutes (b). Error bars represent the SD of 3 independent experiments and alphabets at error bars represent significance ($p<0.05$) among the samples.

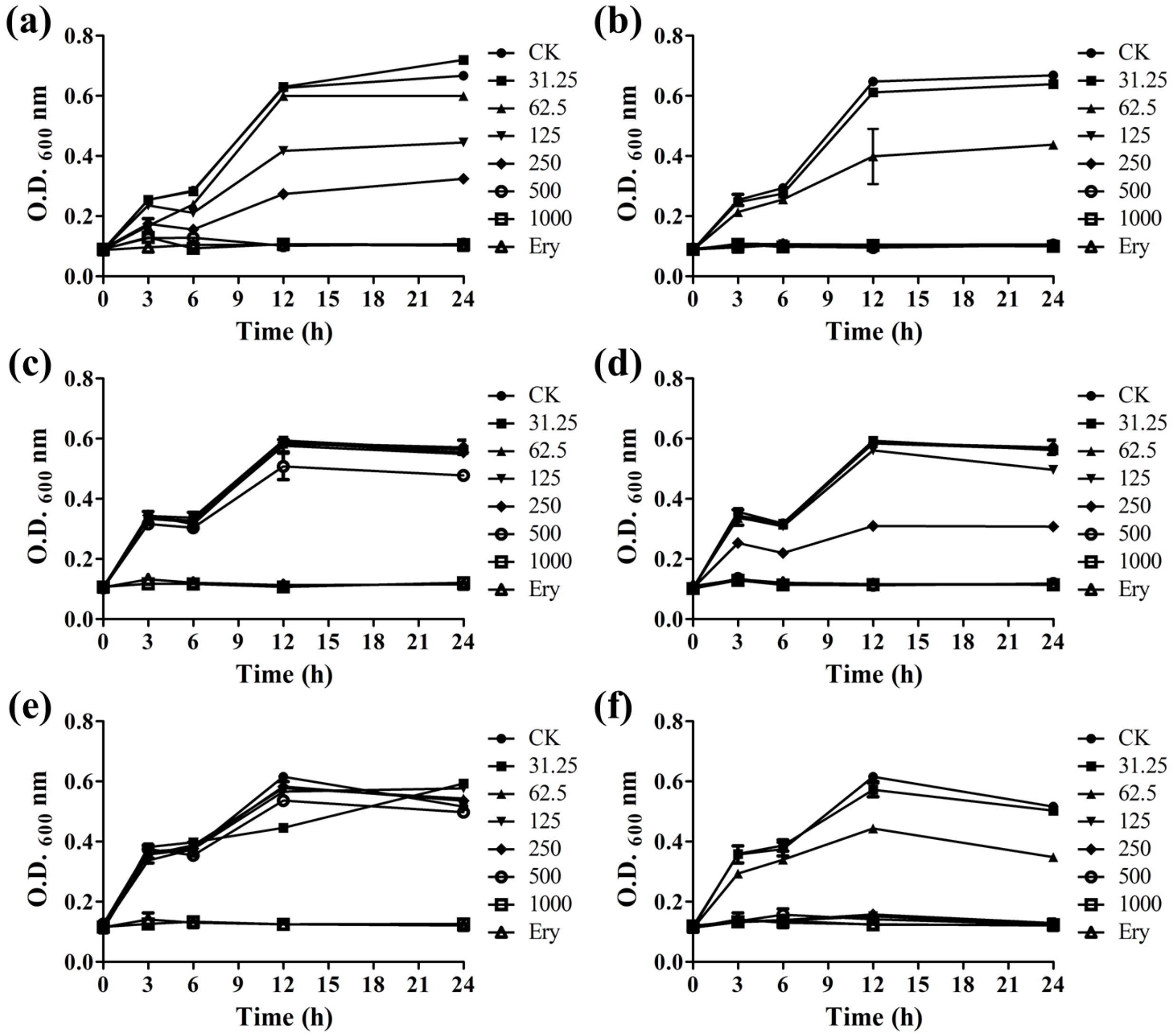

Figure S5. Determination of minimum inhibitory concentration (MIC) of EAE-PL and EAE-PR against Gram-positive pathogens **(a-f)** in microtiter assay (numbers 31.25-1000, represent the treatment concentration in µg/mL). Where, MIC of EAE-PL **(a)** and EAE-PR **(b)** were tested against *B. cereus* cells. Similarly, MIC of EAE-PL **(c)** and EAE-PR **(d)** were tested against *S. aureus* cells and the MIC of EAE-PL **(e)** and EAE-PR **(f)** were tested against *L. monocytogenes* cells. Where, untreated bacterial cells (CK) and antibiotic erythromycin (Ery) treated (100 µg/mL) cells were considered as experimental negative and positive control, respectively.

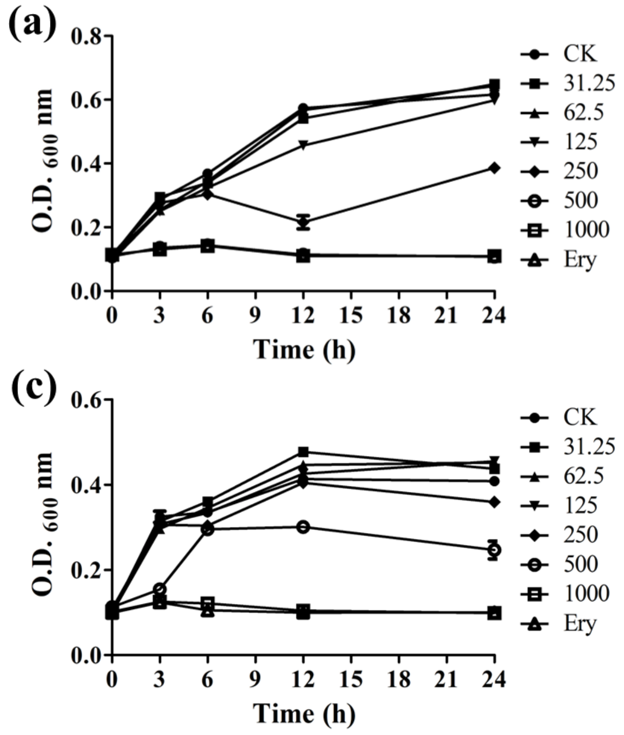

Figure S6. Determination of minimum inhibitory concentration (MIC) of EAE-PL and EAE-PR against Gram-negative pathogens **(a-d)** in microtiter assay (numbers 31.25-1000, represent the treatment concentration in µg/mL). Where, MIC of EAE-PL **(a)** and EAE-PR **(b)** were tested against *E. coli* cells. Similarly, MIC of EAE-PL **(c)** and EAE-PR **(d)** were tested against *S. enterica* cells. Where, untreated bacterial cells (CK) and antibiotic erythromycin (Ery) treated (100 µg/mL) cells were considered as experimental negative and positive control, respectively.

Table S1. The analysis of pharmacokinetic properties of the tentatively identified compounds from EAE-PL using the Lipinski's rule of five through SwissADME web tool.

Compounds	Molecular weight	H-bond		Log P	Molar refractivity	Rules violated
		Donor	Acceptor			
Nicotinic Acid	123.11	1	3	0.86	31.2	0
Succinic anhydride	100.07	0	3	0.62	20.71	0
Myrcene	136.23	0	0	2.89	48.76	0
2-Methoxyresorcinol	140.14	2	3	1.58	36.98	0
Sorbic Acid	112.13	1	2	1.46	31.78	0
Dihydro-beta-tubaic acid	236.26	1	4	2.12	63.70	0
Trigonelline	137.14	0	2	-3.11	35.05	0
Sinapaldehyde	208.21	1	4	1.97	56.55	0
Radicinin	236.22	1	5	1.81	60.42	0
Acetophenone	120.15	0	1	1.64	36.64	0
Vanillyl alcohol	154.16	2	3	1.79	41.08	0
p-Hydroxyphenylacetic acid	152.15	2	3	0.88	40.01	0
Penicillic acid	170.16	1	4	1.16	42.68	0
3-Phenylpropionic acid	150.17	1	2	1.50	42.79	0
Phenylacetic acid	136.15	1	2	1.23	37.99	0
Valacyclovir	324.34	3	7	0.78	82.54	0
Ferulic acid	194.18	2	4	1.62	51.63	0
Eugenol	164.20	1	2	2.37	49.06	0
Epicatechin	290.27	5	6	1.47	74.33	0
Butylphthalide	190.24	0	2	2.53	54.99	0

Table S2. The analysis of pharmacokinetic properties of the tentatively identified compounds from EAE-PR using the Lipinski's rule of five through SwissADME web tool.

Compounds	Molecular weight	H-bond		Log P	Molar refractivity	Rules violated
		Donor	Acceptor			
Salicyluric acid	195.17	3	4	1.16	48.04	0
Limonene	136.23	0	0	2.72	47.12	0
Thiophene A	196.27	0	0	3.45	61.91	0
Zeatin	219.24	3	4	1.04	60.91	0
Sorbic Acid	112.13	1	2	1.46	31.78	0
3-Indoleacetic acid	175.18	2	2	1.01	49.84	0
8-Hydroxyquinoline	145.16	1	2	1.65	43.77	0
Maculosin	260.29	2	3	1.76	76.78	0
Dehydroacetic acid	168.15	0	4	1.08	39.67	0
Esculetin	178.14	2	4	1.25	46.53	0
Foetisulfide A	224.41	0	1	2.63	63.56	0
Haematommic acid	196.16	3	5	0.02	47.80	0
p-Coumaric acid	164.16	2	3	0.95	45.13	0
Vanillin	152.15	1	3	1.57	40.34	0
Scopoletin	192.17	1	4	1.86	51.00	0
Curvulinic acid	210.18	3	5	0.25	52.23	0
Sinapic acid	224.21	2	5	1.63	52.12	0
Dehydrochorismic Acid	224.17	3	6	0.81	52.83	0
Harmine	212.25	1	2	2.07	37.91	0
4-Methoxycinnamic acid	178.18	1	3	1.68	49.60	0
Ethylparaben	166.17	1	3	1.93	44.55	0
Fraxetin	208.17	2	5	1.52	53.02	0
Sinapaldehyde	208.21	1	4	1.97	56.55	0
Ferulic acid	194.18	2	4	1.62	51.63	0
Isofraxidin	222.19	1	5	1.96	57.49	0
Phenylacetic acid	136.15	1	2	1.23	37.99	0

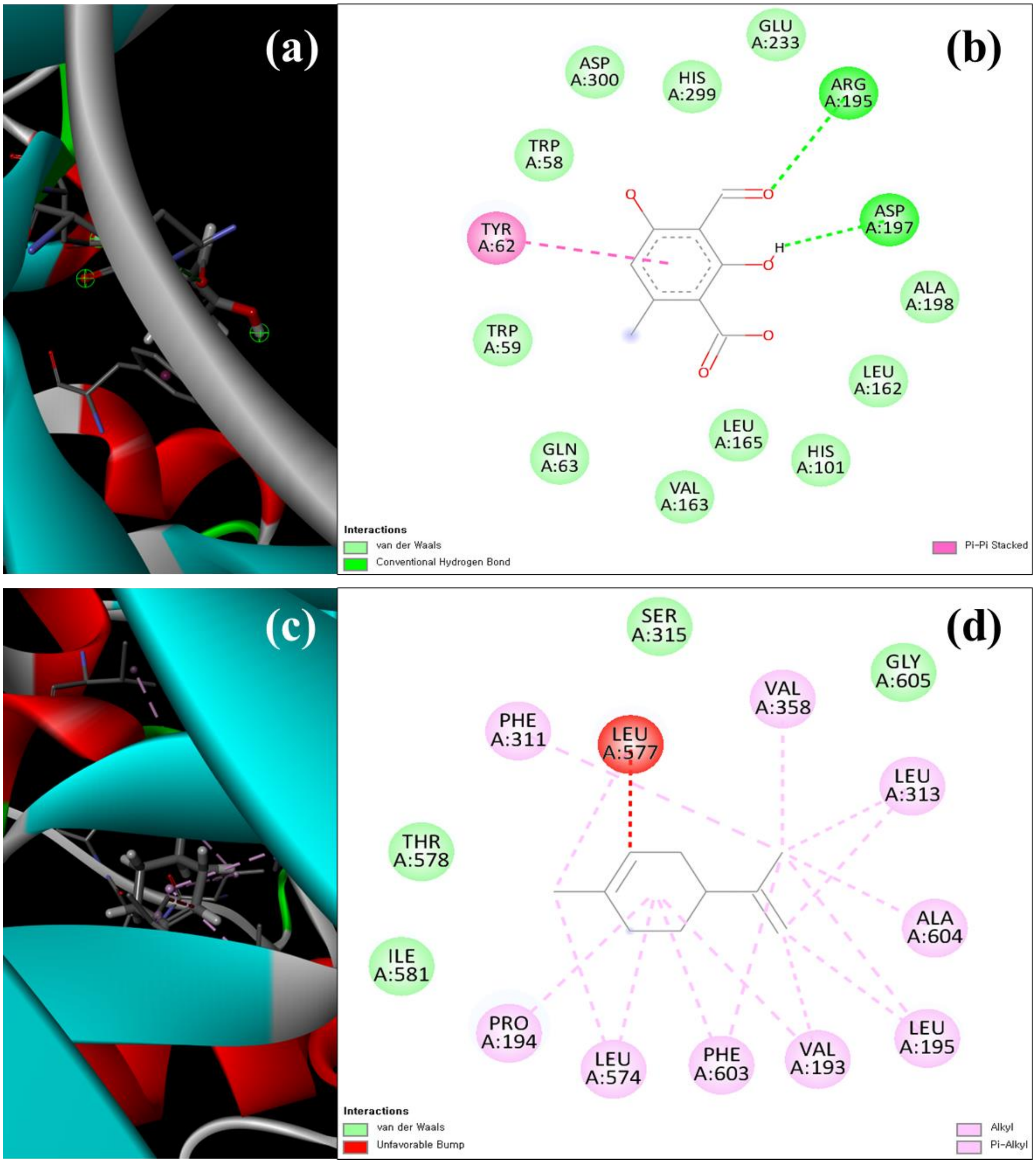

Figure S7. The structure overlay and 3D (a&c), and 2D (b&d) docking simulation of haematommic acid with the crystal structure of α-amylase (a&b) and limonene with α-glucosidase (c&d) during *in-silico* molecular interaction analysis. Where, 3D simulations represent target proteins (thick tube), ligand (thin tube) and interaction forces/bonds (dotted tubes).

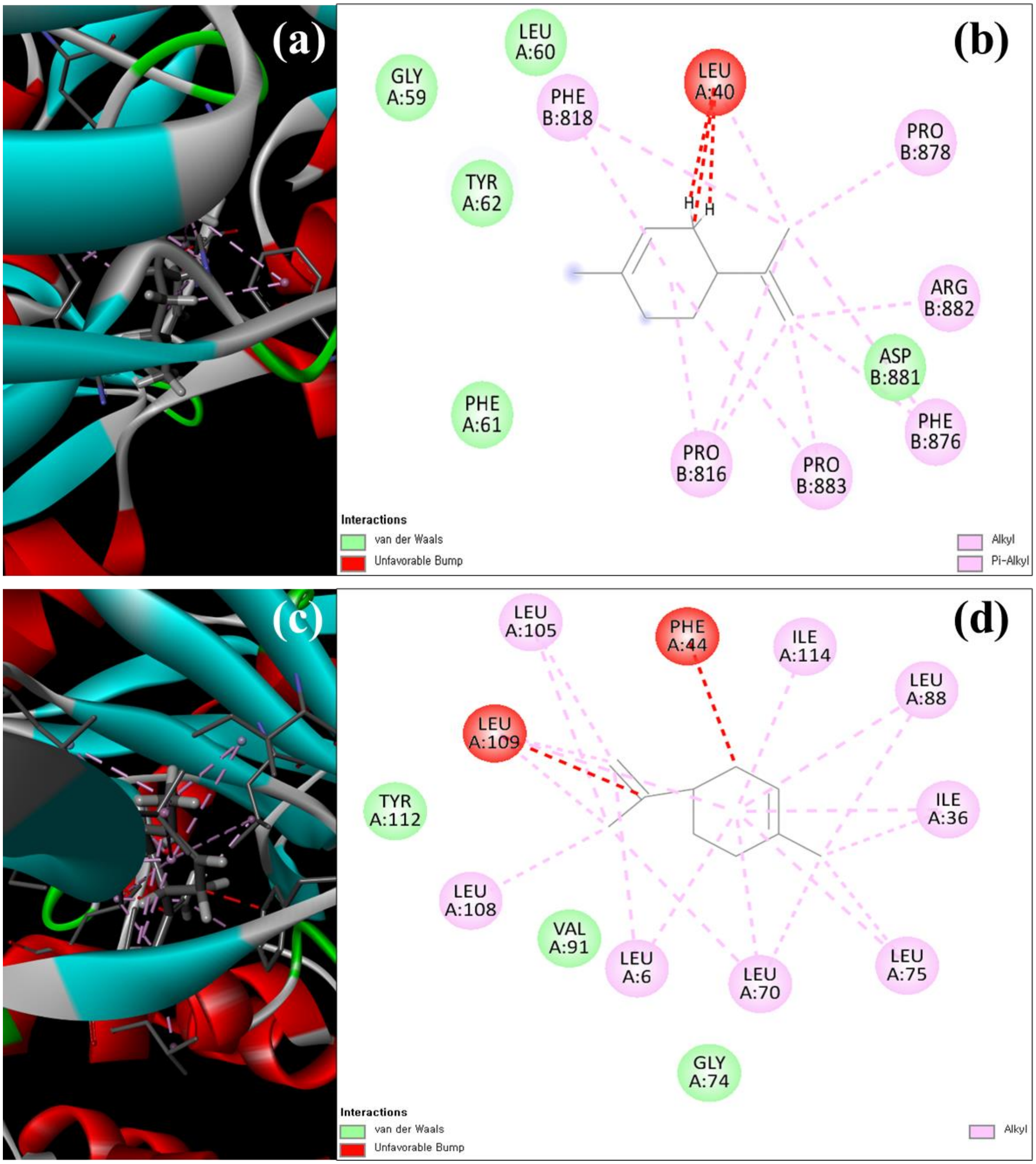

Figure S8. The structure overlay and 3D (a&c), and 2D (b&d) docking simulation of limonene with the crystal structure of NADPH-oxidase (a&b) and D-alanine D-alanine ligase (c&d) during *in-silico* molecular interaction analysis. Where, 3D simulations represent target proteins (thick tube), ligand (thin tube) and interaction forces/bonds (dotted tubes).

Table S3. Summary of the comprehensive hydrogen bonding and Van der Waals interaction with involved amino acids within the binding pockets of α-amylase (PDB: 1OSE) and α-glucosidase (PDB: 5NN8) against the active compounds of EAE-PR during the *in-silico* molecular docking analysis.

Compounds	α-amylase (PDB: 1OSE)		α-glucosidase (PDB: 5NN8)	
	H-bond (amino acids) / C-H bond (amino acids)	Van der Waals interaction (amino acids)	H-bond (amino acids) / C-H bond (amino acids)	Van der Waals interaction (amino acids)
3-Indoleacetic acid	3 (PHE315, ARG343, TRP388) / -	2 (TRP316, GLU385)	- / -	5 (LEU701, ALA704, GLN776, ILE780, VAL816)
4-Methoxycinnamic acid	2 (GLN63, ASP300) / -	4 (LEU165, ARG195, HIS305)	5 (TRP481, ASP518, MET519, ARG600, LEU677) / -	7 (TRP376, ASP404, ASP616, LEU650, ASN675, SER676, SER679)
8-Hydroxyquinoline	- / 1 (LYS68)	5 (GLU76, ASP181, TYR182, LEU183, ILE189)	1 (GLN682) / -	9 (PHE371, PHE396, CYS658, ASN673, GLU683, PHE687, GLN692, ARG696, ALA698)
Curvulinic acid	2 (ALA128, LYS185) / 1 (TYR182)	5 (LYS68, LEU69, VAL129, ASP181, LEU186)	2 (ASP774, LEU775) / -	10 (GLN663, PHE667, LYS697, ALA698, GLN776, VAL778, ILE780, THR813, ASN815, VAL816)
Dehydroacetic acid	1 (SER270) / -	4 (THR264, TRP269, ALA310, SER311)	1 (ARG600) /-	13 (ARG375, ASP404, LEU405, ILE441, TRP481, TRP516, ASP518, TRP613, ASP616, ARG676, ASN675, SER676, LEU677)
Dehydrochorismic Acid	2 (GLN63, HIS299) / -	15 (TRP58, TRP59, GLU60, TYR62, VAL98, HIS101, LEU162, VAL163, LEU165, ARG195, ASP197, ALA198, GLU233, ASP300, HIS305)	2 (ASP645, ARG672) / 1 (HIS674)	9 (TRP376, ASP404, ILE441, TRP481, ASP518, GLY615, ASN675, SER676, LEU677)
Esculetin	2 (ARG343, VAL383) / -	1 (ALA318)	3 (ARG600, TRP613, ASP616) / -	12 (TYR292, TRP376, TRP481, ASP518, MET519, GLY615, ASP645, ARG672, HIS674, ASN675, SER676, LEU677)
Ethylparaben	1 (MET202) / -	5 (ALA97, LEU170, ILE179, SER199, ASP206)	1 (ARG385) / -	8 (THR384, VAL387, GLU389, GLN401, VAL428, GLU430, GLY434, TYR438)

Ferulic acid	2 (SER270, SER311) / -	5 (THR264, TRP269, GLY308, GLY309, ILE312)	3 (ARG600, ASN675, LEU677)	11 (ASP282, ARG375, ASP404, TRP481, TRP516, ASP518, MET519, ASP616, HIS674, SER676, LEU678)
Foetisulfide A	- / -	8 (GLU60, GLN63, VAL163, LEU165, ARG195, ASP197, ASP300, HIS305)	- / -	8 (THR700, ALA704, LEU750, GLN776, LEU818, ILE824, LEU826, ALA844)
Fraxetin	2 (LYS35, ARG392) / -	5 (PRO34, ASN393, ASP456, GLU493, SER494)	3 (TRP376, ASP518, MET519) / 1 (ASP616)	9 (ASP282, ARG375, ILE441, TRP516, TRP613, HIS674, ASN675, SER676, LEU677)
Haematommic acid	2 (ARG195, ASP197) / -	11 (TRP58, TRP59, GLN63, HIS101, LEU162, VAL163, LEU165, ALA198, GLU233, HIS299, ASP300)	3 (ARG594, LEU868, GLU869) / -	5 (MET363, PHE858, ASP860, LEU865, GLU866)
Harmine	- / 1 (VAL383)	4 (ASP317, LYS322, ARG343, ARG346)	- / 1 (ASP518)	9 (ASP404, ILE441, ARG600, TRP613, ASP616, ASN675, SER676, LEU677, LEU678)
Isofraxidin	1 (LYS35) / 1 (ASP456)	8 (PRO34, ARG392, ASN393, VAL452, LYS457, VAL452, HIS491, GLU493)	1 (SER864) / -	6 (PRO595, HIS714, PHE858, ASP860, GLU866, GLU869)
Limonene	- / -	7 (GLU60, ARG61, GLN63, GLY104, VAL163, ASP300, HIS305)	- / -	4 (SER315, THR578, ILE581, GLY605)
Maculosin	3 (ALA318, ARG343, GLU390) / -	3 (ASP317, LYS322, VAL383)	2 (ASP282, ARG600) / -	5 (LEU405, ASP518, SER523, ASP616, PHE649)
p-Coumaric acid	2 (GLN63, ASP197) / -	5 (TRP58, TRP59, GLU60, LEU165, HIS299)	- / -	5 (LEU701, HIS708, ALA749, ILE780, LEU818)
Phenylacetic acid	1 (ARG343) / -	3 (ALA318, GLU385, TRP388)	1 (THR234) / 1 (GLY219)	6 (LEU152, PHE181, VAL220, THR235, ALA237, VAL339)
Salicyluric acid	1 (CYS378) / 1 (VAL383)	6 (TRP316, ALA318, THR377, TRP382, CYS384, GLU385)	4 (TYR292, ASP404, ARG600, TRP613) / 2 (ASP518, PHE649)	8 (TRP481, TRP516, GLY615, HIS674, ASN675, SER676, LEU677, LEU678)
Scopoletin	- / 2 (TRP316, VAL383)	4 (ALA318, THR377, CYS378, TRP382)	1 (ARG600) / 2 (ASP518, ASP616)	10 (TYR292, TRP376, LEU405, TRP481, TRP516, SER523, TRP613, GLY615, SER676, LEU677)
Sinapaldehyde	- / -	7 (TRP58, TYR62, GLN63, VAL163, ARG195, ASP197, ASP300)	1 (LEU677) / 2 (ASP518, ASP616)	9 (TRP376, ASP443, MET519, ARG600, ASP645, LEU650,

				ARG672, SER676, LEU678)
Sinapic acid	4 (THR264, SER270, GLY308, SER311) / -	3 (LYS261, TRP269, GLY309)	2 (ASP404, LEU678) / -	8 (TRP376, ILE441, TRP481, TRP516, ASP616, PHE649, HIS674, ASN675)
Sorbic Acid	- / -	8 (ILE179, LEU183, PHE194, ILE196, ASP206, LEU211, LEU214, ILE230)	2 (ARG412, THR415) / -	6 (TYR407, MET408, ASP413, MET440, ILE441, PHE506)
Thiophene A	- / -	11 (TRP58, TRP59, GLU60, GLN63, GLY104, SER105, GLY106, GLY164, ARG195, ASP197, ASP300)	- / -	3 (THR700, ALA704, GLN776)
Vanillin	1 (TYR67) / 1 (ALA128)	4 (SER66, LYS68, ILE179, ASP181)	- / 2 (ASP518, ASP616)	9 (TRP376, ILE441, MET519, ARG600, PHE649, HIS674, ASN675, SER676, LEU677)
Zeatin	4 (THR6, ARG398, ASP402, ARG421) / 2 (PRO332, GLY334)	7 (ALA3, ARG10, THR11, ARG252, THR336, GLU404, VAL401)	1 (HIS717) / 1 (GLU869)	9 (MET363, TYR710, HIS714, PHE858, ASP860, SER864, LEU865, GLU866, ARG870)
Acarbose	2 (GLY304, GLY306) / -	9 (TRP58, TRP59, LEU162, VAL163, LEU165, HIS305, ALA307, GLY309, ALA310)	3 (HIS507, ASP508, THR593) / 3 (GLN509, ARG591, GLU863)	8 (PRO425, HIS432, ARG437, VAL510, ASP513, ALA590, GLY592, SER864)

Table S4. Summary of the comprehensive hydrogen bonding and Van der Waals interaction with involved amino acids within the binding pockets of NADPH-oxidase (PDB: 2CDU) and D-alanine D-alanine ligase (PDB: 2PVP) against the active compounds of EAE-PR during the *in-silico* molecular docking analysis.

Compounds	NADPH-oxidase (PDB: 2CDU)		D-alanine D-alanine ligase (PDB: 2PVP)	
	**H-bond (amino acids) / C-H bond (amino acids)**	**Van der Waals interaction (amino acids)**	**H-bond (amino acids) / C-H bond (amino acids)**	**Van der Waals interaction (amino acids)**
3-Indoleacetic acid	1 (LYS17) / -	4 (HIS10, THR13, PHE14, PHE884)	2 (GLU451, TYR465) / GLU203	3 (ASN129, LEU469, ASP590)
4-Methoxycinnamic acid	3 (TYR159, ILE160, SER326) / TYR188	9 (GLY158, GLY161, CYS242, GLY244, PHE245, ILE297, LEU299, SER328, SER339)	4 (GLU577, LYS581, ASP626, ASP627) / 1 (THR631)	5 (PHE558, SER559, TYR560, LEU624, PHE628)
8-Hydroxyquinoline	1 (ARG431) /-	6 (GLU366, PHE367, HIS461, GLY510, LEU511, TYR513)	2 (PRO208, ILE210) / -	3 (VAL183, GLU215, ILE292)
Curvulinic acid	3 (THR742, SER744, LEU797) / -	6 (ALA746, TYR747, ILE748, ALA798, ASN801, LEU803)	2 (ILE210, ASN299) / -	7 (LEU145, LYS171, GLU207, PRO208, PHE209, VAL213, GLU215)
Dehydroacetic acid	1 (PHE884) / 1 (HIS10)	5 (THR9, THR13, LYS17, LEU40, PRO883)	2 (LYS329, LEU419) / -	7 (PHE332, PRO418, ALA44, ALA636, LEU639, MET641, GLU643)
Dehydrochorismic Acid	3 (ILE611, ILE694, GLY695) / -	8 (PRO568, THR569, CYS584, GLY609, TRY610, GLY612, TYR639, LEU692)	5 (SER277, ASP278, LEU279, ASN458, TYR460) / -	6 (GLU122, LEU126, TYR136, LEU140, LEU464, GLU532)
Esculetin	4 (SER777, SER790, GLY792, ASN794) / -	5 (PHE640, ILE748, PRO749, LEU750, LEU797)	1 (ILE210) / -	7 (LEU145, LYS171, GLU207, PRO208, PHE209, VAL213, ASN299)
Ethylparaben	1 (PHE433) / -	6 (ARG431, ILE438, GLN441, THR460, HIS461, THR464)	1 (TYR385) / -	8 (ASP368, GLU369, TYR374, ILE376, PHE386, LYS390, LYS392, LYS393)
Ferulic acid	3 (TYR610, ILE611, SER777) / 1 (SER777)	6 (GLY609, TYR639, PHE696, PRO749, SER778, SER779)	1 (LEU461) / -	9 (TYR131, LEU135, GLU203, GLU451, THR462, LEU464, TYR465, ASP590, SER589)
Foetisulfide A	- / -	7 (PRO365, PHE425, ARG431, THR464, GLY510, LEU511, PHE512)	2 (TYR465, SER589) / -	6 (ASN129, TYR136, GLU451, ASP468, LEU469, ASP590)

Fraxetin	1 (TYR288) / 1(TYR296)	6 (TYR188, THR291, SER326, GLY341, ASN343, ASN350)	3 (GLU122, SER277, ASN458) / -	5 (LEU140, LYS274, ASP278, VAL454, TYR460)
Haematommic acid	1 (HIS10) / -	6 (THR9, THR13, GLN18, VAL304, PRO883, PHE884)	1 (ASN494) / -	5 (ASP475, TYR476, MET491, GLU536, PRO537)
Harmine	1 (HIS10) / 1(HIS10)	4 (THR9, THR13, GLN18, LEU40)	- / 3 (GLU536, VAL542, LYS500)	6 (LEU474, SER507, PRO537, GLN540, GLY541, GLU544)
Isofraxidin	2 (SER777, SER779) / 2 (SER790, GLY792)	5 (LYS638, ILE748, THR791, ASN794, ILE793)	1 (TYHR473) / 3 (ILE471, THR476, ASP475)	5 (ASP139, LEU464, ALA466, LYS472, LEU474)
Limonene	- / -	5 (GLY59, LEU60, PHE61, TYR62, ASP881)	- / -	3 (GLY74, VAL91, TYR112)
Maculosin	1 (LYS17) / -	5 (HIS10, THR13, LEU40, ARG882, PHE884)	2 (THR473, ASP475) / 2 (THR473, TYR476)	5 (ASP139, LEU464, LYS472, LEU474, TYR476)
p-Coumaric acid	3 (TYR610, ILE611, SER777) / 1 (SER779)	4 (GLY609, PHE696, ILE748, SER790)	1 (ASN494) / -	6 (VAL477, LEU479, ALA487, LEU488, ASN492, PHE497)
Phenylacetic acid	1 (ARG431) / -	6 (GLU366, PHE367, PHE425, PRO427, LEU511, TYR513)	1 (CYS333) / -	8 (ILE365, LEU367, LEU404, LEU417, VAL420, LEU437, TYR441, ILE443)
Salicyluric acid	2 (GLU366, PHE367) / -	9 (PHE425, ARG431, PRO432, HIS461, LYS504, GLY510, LEU511, PHE512, TYR513)	2 (HIS372, TYR374) / -	9 (PHE366, ASP368, ASN370, ILE376, TYR385, PHE386, LYS390, LYS392, LYS393)
Scopoletin	- / -	9 (GLY609, ILE611, LEU636, ILE748, LEU750, SER777, SER779, SER790, ASN794)	- / 1 (LEU488)	7 (LEU479, ALA487, PHE493, PHE495, PRO496, GLU536, PHE538)
Sinapaldehyde	1 (GLN444) / 2 (GLN444, ASP772)	6 (ASP416, ALA419, ARG759, LEU760, LYS771, GLY774)	3 (ASN129, GLU203, SER589) / 1 (GLU451)	7 (LEU132, LEU135, LEU455, LEU464, TYR465, LEU469, ASP590)
Sinapic acid	4 (TYR610, ILE611, SER777, SER779) / 1 (PRO749)	6 (GLY609, GLY612, GLY695, PHE696, ASN753, SER790)	2 (ASN129, GLU203) / 2 (GLU203, GLU451)	10 (LEU132, LEU135, ASN151, LEU455, LEU461, LEU464, TYR465, LEU469, SER589, ASP590)
Sorbic Acid	1 (ARG431) / -	4 (GLU366, ASP430, PRO432, TYR513)	1 (CYS333) / -	8 (PHE111, LEU335, LEU367, LEU417, VAL420, ILE421, LEU438, ILE443)
Thiophene A	- / -	6 (HIS10, PHE14, GLY59, ARG308, PRO816, ARG882)	- / -	5 (ASN129, GLU203, GLU451, ASP590, SER589)

Vanillin	2 (ARG308, MET322) / -	4 (LEU309, LYS320, ASP321, ASP867)	1 (TYR385) / 1 (LYS390)	4 (PHE366, ASP368, ILE376, LYS392)
Zeatin	1 (PRO749) / 1 (PHE696)	10 (SER492, SER566, LYS585, GLY609, TYR639, GLY695, ASP733, TYR747, ILE748, LEU750)	2 (GLU215, SER178) / -	6 (LEU145, VAL181, VAL183, PRO208, VAL213, PHE290)
Ascorbic acid	1 (ASP733) / -	13 (GLY458, CYS459, HIS461, ALA462, GLY463, THR564, GLY565, SER566, LYS585, GLY732, SER734, ALA751, ALA754)	ND	ND
Erythronolide A (Erythromycin)	ND	ND	2 (ASN236, GLU215) / -	7 (LYS171, SER178, PHE209, ILE210, GLN211, GLY212, ASN299)
